# Cell and context-dependent sorting of neuropathy-associated protein NDRG1 – insights from canine tissues and primary Schwann cell cultures

**DOI:** 10.1186/s12917-019-1872-2

**Published:** 2019-04-27

**Authors:** Fredrik S. Skedsmo, Michael A. Tranulis, Arild Espenes, Kristian Prydz, Kaspar Matiasek, Gjermund Gunnes, Lene C. Hermansen, Karin H. Jäderlund

**Affiliations:** 1 Department of Companion Animal Clinical Sciences, Norwegian University of Life Sciences, Oslo, Norway; 2 Department of Basic Sciences and Aquatic Medicine, Norwegian University of Life Sciences, Oslo, Norway; 3 Department of Biosciences, University of Oslo, Oslo, Norway; 4 Section of Clinical & Comparative Neuropathology, Centre for Clinical Veterinary Medicine, Ludwig-Maximilians-Universität, Munich, Germany; 5 Department of Plant Sciences, Norwegian University of Life Sciences, Ås, Norway

**Keywords:** Polyneuropathy, Charcot-Marie-tooth disease (CMT), Dog, Greyhound, Alaskan malamute, Microtubules, Microtubule-associated protein (MAP), Myelin

## Abstract

**Background:**

Mutations in the *N-myc downstream-regulated gene 1* (*NDRG1*) can cause degenerative polyneuropathy in humans, dogs, and rodents. In humans, this motor and sensory neuropathy is known as Charcot-Marie-Tooth disease type 4D, and it is assumed that analogous canine diseases can be used as models for this disease. NDRG1 is also regarded as a metastasis-suppressor in several malignancies. The tissue distribution of NDRG1 has been described in humans and rodents, but this has not been studied in the dog.

**Results:**

By immunolabeling and Western blotting, we present a detailed mapping of NDRG1 in dog tissues and primary canine Schwann cell cultures, with particular emphasis on peripheral nerves. High levels of phosphorylated NDRG1 appear in distinct subcellular localizations of the Schwann cells, suggesting signaling-driven rerouting of the protein. In a nerve from an Alaskan malamute homozygous for the disease-causing *Gly98Val* mutation in *NDRG1*, this signal was absent. Furthermore, NDRG1 is present in canine epithelial cells, predominantly in the cytosolic compartment, often with basolateral localization. Constitutive expression also occurs in mesenchymal cells, including developing spermatids that are transiently positive for NDRG1. In some cells, NDRG1 localize to centrosomes.

**Conclusions:**

Overall, canine NDRG1 shows a cell and context-dependent localization. Our data from peripheral nerves and primary Schwann cell cultures suggest that the subcellular localization of NDRG1 in Schwann cells is dynamically influenced by signaling events leading to reversible phosphorylation of the protein. We propose that disease-causing mutations in *NDRG1* can disrupt signaling in myelinating Schwann cells, causing disturbance in myelin homeostasis and axonal-glial cross talk, thereby precipitating polyneuropathy.

## Background

The *N-myc downstream-regulated gene 1* (*NDRG1*) was first described as a gene that is up-regulated by homocysteine [1] and during cellular differentiation [2], and later identified as the mutated gene in an inherited demyelinating neuropathy, Charcot-Marie-Tooth type 4D (CMT4D), in humans [3]. Subsequently, mutations in *NDRG1* were observed in Greyhound show dogs [4] and Alaskan malamutes [5] suffering from inherited peripheral neuropathy. *NDRG1* encodes a 43-kDa protein in humans, which is expressed in many tissues, predominantly epithelial cells [6]. High levels of NDRG1 have been found in human and murine peripheral nerves, where the protein was expressed in the myelinating Schwann cells [7] and constituted 0.09% of total myelin proteins in the peripheral nervous system [8].

Furthermore, NDRG1 expression is downregulated in several malignancies, for instance those originating from the prostate and colon in humans [2, 9–13]. Decreased NDRG1 levels in these neoplasms is associated with a poor prognosis [9, 11–13], possibly explained by the ability of NDRG1 to inhibit epithelial-mesenchymal transition (EMT) [14]. At the molecular level, NDRG1 has been linked to vesicular transport [15], as a Rab4a-effector involved in recycling of E-cadherin [16] and being involved in the uptake of low-density lipoproteins (LDL) [17]. In line with the wide range of reported functions, NDRG1 can undergo substantial post-translational modifications by proteolytic cleavage [18], SUMO 2/3-modification [19] and phosphorylation [20–22].

Despite the ubiquitous expression of NDRG1 in the epithelium of different tissues, the pathologic changes reported from humans, rodents, and dogs with *NDRG1*-associated neuropathies are restricted to the peripheral nervous system [3–5, 7], and, to a lesser degree, the central nervous system [23]. This suggests that a comparative study of NDRG1 in different cell types from dogs with and without mutation in NDRG1 is required to understand the many facets of this protein.

In both humans and mice with *NDRG1* mutations, the degeneration of the nerves is described as a primary demyelination [24]. In contrast, the polyneuropathies of Greyhounds and Alaskan malamutes were dominated by axonal changes [4, 5]. Greyhounds, humans and mice with *NDRG1* mutations all have a total NDRG1 deficiency [24], suggesting that NDRG1 is involved in axonal-glial cross talk and that disruption of NDRG1 function may affect either side of the communication axis. A detailed mapping of the cellular and subcellular distribution of NDRG1, as well as post-translational modifications of the protein in peripheral nerves of dogs, is one prerequisite for deciphering NDRG1’s roles in neuropathies. Studies of NDRG1 in the highly specialized Schwann cells can also have broader implications and contribute to our understanding of NDRG1 in other tissues during physiological conditions, as well as in malignancies.

In comparison with laboratory rodents, dogs offer significant advantages as models for human diseases. Dogs have a life expectancy and body size more similar to humans [4], and, as companion animals, they are exposed to the same environmental factors as their human counterparts. In addition, they have naturally occurring *NDRG1* mutations. Thus, the aim of this study was to describe and interpret the immunolocalization of NDRG1 isoforms in tissues and cells from control dogs and an Alaskan malamute dog homozygous for a disease-causing *Gly98Val* mutation in *NDRG1* (hereafter called *NDRG1*^mut/mut^ Alaskan malamute). The results of this should aid in our understanding of *NDRG1*-associated diseases in dogs, humans, and rodents.

## Results

### Levels of NDRG1 isoforms vary significantly between tissues

**Fig. 1:**
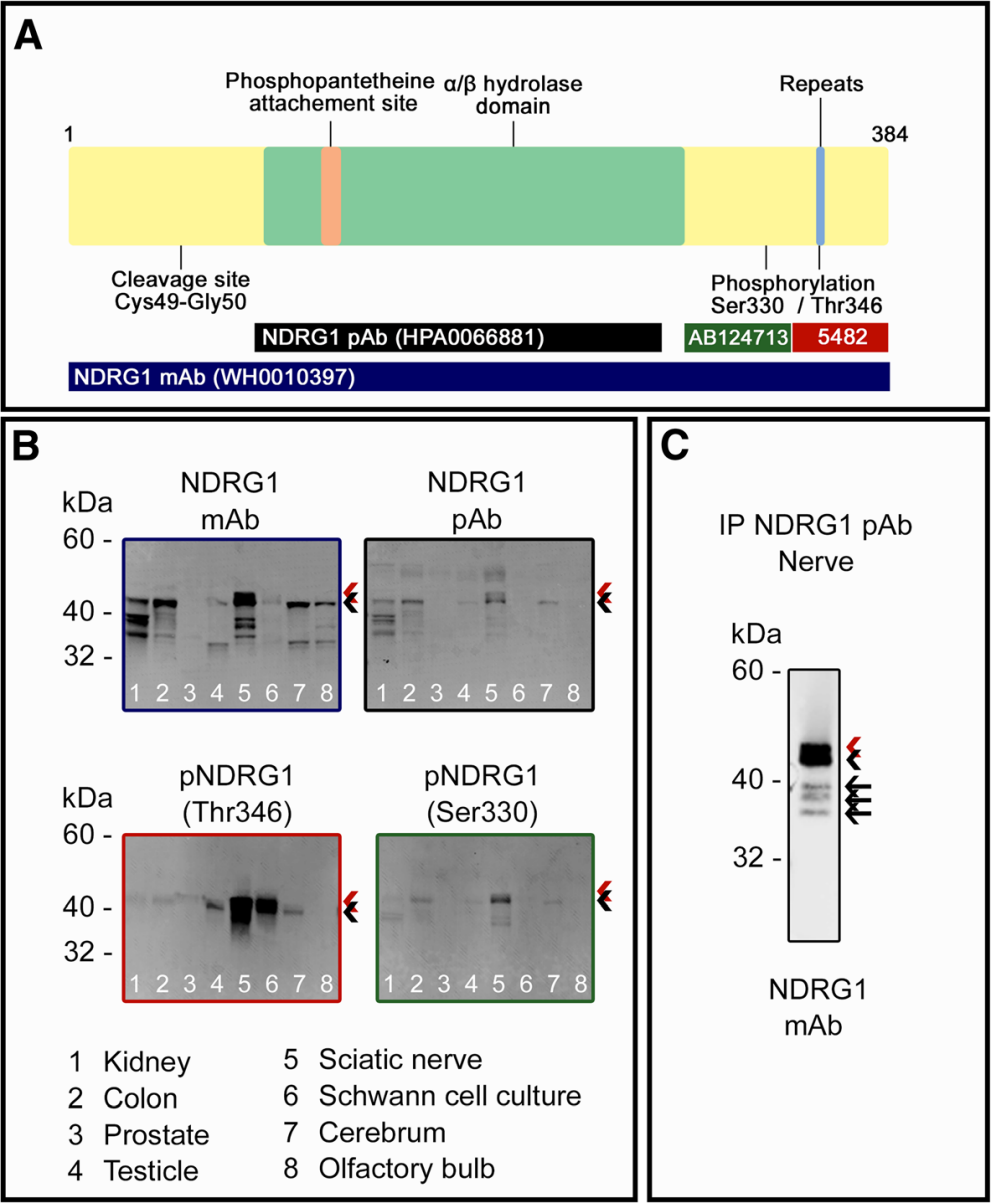
Western blot analysis of canine tissues from control dogs. **a** Schematic structure of the canine NDRG1 protein and the epitopes of the NDRG1 antibodies used in the analyses. Note that the epitope of the NDRG1 mAb is not known. **b** Western blotting of lysate from canine tissues. **c** Western blotting after immunoprecipitation from peripheral nerve lysate. Full-length protein and phosphorylated protein are indicated by black and red arrowhead, respectively. In C, black arrows mark the truncated isoforms

Western blotting with four antibodies recognizing different epitopes of NDRG1 revealed several isoforms and dissimilarities between the analyzed tissues. The schematic structure of the protein and the antibody epitopes are summarized in Fig. 1a. The 42 kDa band, corresponding to the canine full-length protein, is recognized by all antibodies (Fig. 1b), albeit with different strengths. One of the phospho-specific antibodies, recognizing phosphorylation at threonine 346 (Thr346), revealed a band with molecular mass of 45–47 kDa, prominently present in nerve tissue preparations, Schwann cell culture, and testicle, but almost undetectable in the other tissue lysates. The reduced electrophoretic mobility of the phosphorylated protein is mainly caused by an increased size and bulkiness compared to the unphosphorylated isoform. Several, but not identical bands of lower molecular mass, ranging from 30 to 37 kDa were present at different levels in all the lysates, including the Schwann cell culture, suggesting that NDRG1 undergoes complex and tissue-specific proteolytic processing and/or degradation. The weak band from the prostate reflects low abundance of NDRG1 compared with the high levels of total protein. However, as shown immunohistochemically (Fig. 3e), there is strong expression of NDRG1 in the prostate.

### Specificity of the antibodies

To ascertain the specificity of the NDRG1 antibodies used, we performed an immunoprecipitation from peripheral nerve lysate with the polyclonal anti-NDRG1 antibody produced in rabbit, and a subsequent Western blot with the monoclonal anti-NDRG1 antibody produced in mouse (Fig. 1c). The presence of bands corresponding to full-length protein, phosphorylated protein, and proteolytically processed NDRG1 with this method, supports that the detection of these NDRG1 isoforms is specific, as they are recognized by both antibodies. The presence of the 45–47 kDa band indicated that the antibodies also recognize the phosphorylated form of the protein to some extent.

**Fig. 2:**
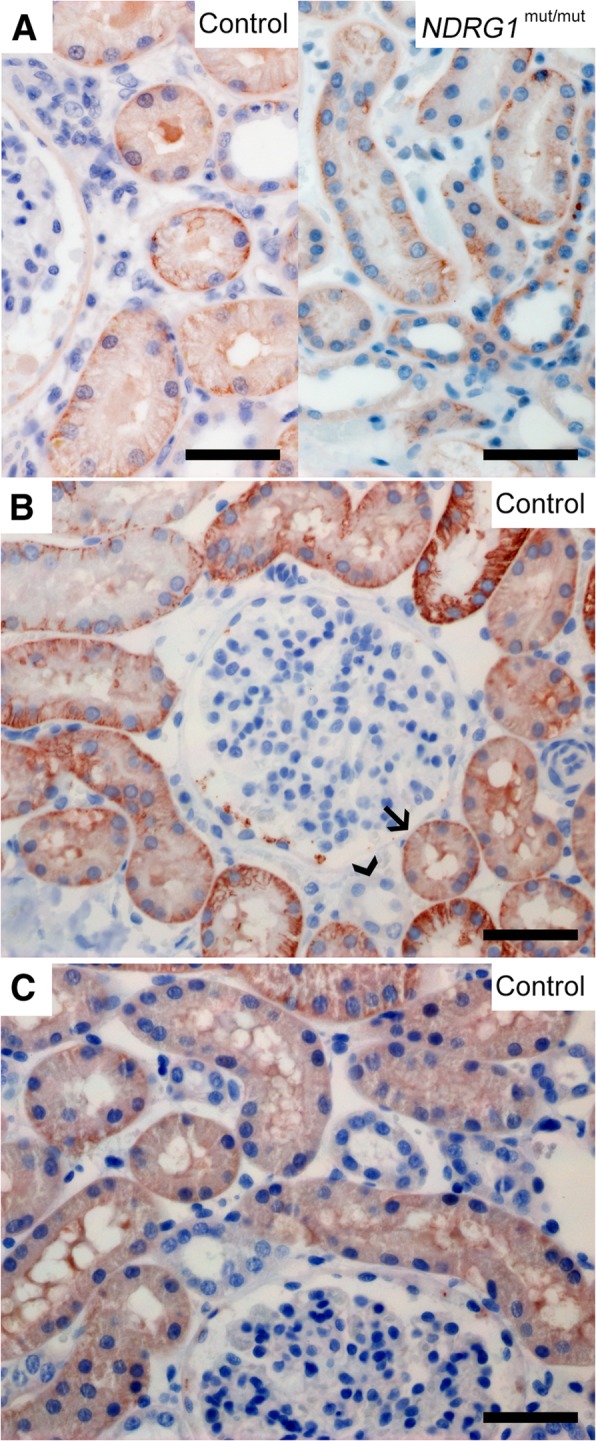
Expression of NDRG1 in the kidney. NDRG1 is expressed in the epithelium of the proximal convoluted tubules in the kidneys (**a-c**). The figure compares the signal from the three different antibodies against NDRG1 used in the immunohistochemical analysis (**a**: mAb produced in mouse, **b**: pAb produced in goat, **c**: pAb produced in rabbit). In A, the NDRG1 signal from the *NDRG1*^mut/mut^ Alaskan malamute is similar to the control. In B, the proximal and distal convoluted tubules are indicated by arrow and arrowhead, respectively. Bar 50 μm

For the immunohistochemical analysis of canine tissues, three different NDRG1 antibodies were used in parallel. The signals from these antibodies were similar, as shown in Fig. 2, indicating a specific detection of NDRG1 by immunohistochemistry. However, the monoclonal anti-NDRG1 antibody produced in mouse (Fig. 2a) consistently yielded a stronger signal than the two others (Fig. 2b-c).

### Immunoreactivity was strong in epithelial cells

**Fig. 3:**
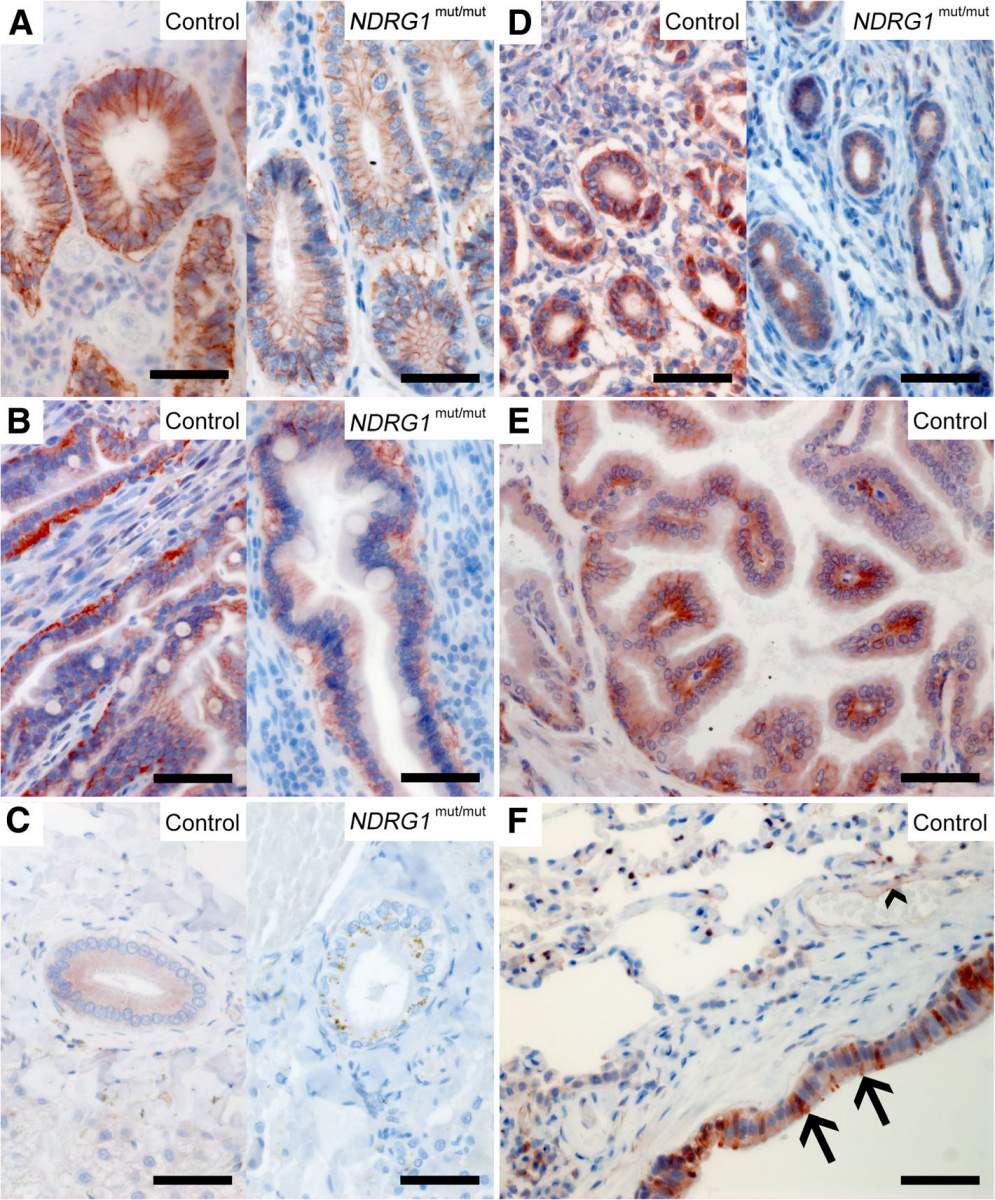
Expression of NDRG1 in epithelia. Strong signal is present in the epithelium of the colon (**a**) and jejunum (**b**) of both the control and *NDRG1*^mut/mut^ Alaskan malamute, whereas NDRG1 staining in the epithelial cells of the bile ducts in the liver was only detected in the control dog (**c**). NDRG1 is also present in the endometrium (**d**) and the epithelium of the prostate (**e**). In the lungs (**f**), the club cells (arrows) of the bronchiolar epithelium display a more intense signal than the surrounding epithelial cells. In addition, signal from the endothelium (arrowhead) can be seen. Note that except for the lack of signal in the bile ducts, the NDRG1 staining in the *NDRG1*^mut/mut^ Alaskan malamute is similar to the controls. The extensive yellow-brown granules in the bile ducts of the *NDRG1*^mut/mut^ Alaskan malamute is interpreted as pigment deposits. Bar 50 μm

Immunohistochemistry of canine tissues showed that epithelial cells have strong expression of NDRG1 protein, in all the investigated digestive, urinary, and reproductive organs. However, the staining pattern differed between the epithelial tissues. The distribution of NDRG1 in all the investigated tissues is summarized in Table 1. In addition to the control dogs, tissues from one *NDRG1*^mut/mut^ Alaskan malamute were immunostained. Micrographs from this dog are shown for some tissues. In general, the staining pattern and intensity were similar to what was observed for the control dog, unless otherwise stated. In the colon (Fig. 3a) and jejunum (Fig. 3b), the epithelium of the mucosa, including the intestinal glands, stained strongly throughout. The immunoreactivity was predominantly localized to the basolateral region. The acinar cells of the exocrine pancreas showed a cytoplasmic staining pattern, which again was most pronounced basolaterally (not shown). In the liver, the hepatocytes did not show any signal, but a granular signal was detectable in the cytoplasm of the bile duct epithelium in the majority of the dogs (Fig. 3c). There was no signal from the bile duct epithelium of the *NDRG1*^mut/mut^ Alaskan malamute. However, as this signal was not consistently present in the control dogs, this could be an incidental finding rather than an effect of the genotype.

**Table 1:** Distribution of NDRG1 protein in canine tissues and cells

Organ	Cell type	Epithelial (E) Mesenchymal (M) Neural (N)	NDRG1 staining pattern			
			Cytoplasmic^a^	Basolateral	Nuclear	Centrosomal
Kidney	Epithelium, proximal tubules	E	+	+		
Jejunum, colon	Epithelium	E		+	+	+
	Smooth muscle cells	M	+			
Lung	Epithelium, bronchioles	E	+	+		
	Club cells	E	+			
Liver	Epithelium, bile ducts	E	+			
Pancreas	Acinar cells	E	+	+	+	+
Ovary	Granulosa cells	E		+		
	Cells of the corpus luteum	E	+			
Uterus	Epithelium of the endometrium	E	+			
	Smooth muscle cells of the myometrium	M	+			
Testicle	Spermatogonia	M			+	
	Spermatids	M	Midpiece			
Prostate	Secretory epithelium	E	+	+	+	+
Lungs	Epithelium of the bronchioles	E	+	+		
Spleen, lymph nodes	Dendritic cells	M	+		+	
	Macrophages in the wall of ellipsoids	M	+			
Blood vessels	Endothelium	E	+			
	Smooth muscle cells	M	+			
Cerebral cortex	Oligodendrocytes	N	+		+	
Cerebellum	Purkinje neurons	N	+			
	Oligodendrocytes	N	+		+	
Spinal cord	Oligodendrocytes	N	+		+	
Peripheral nerves	Schwann cells	N	+		+	
Schwann cell culture		N	+		+	+

^a^The cytoplasmic staining was mostly diffuse, but in some tissues, a distinct granular pattern was observed, such as the bile ducts and cells of the corpus luteum

Note: All the different cell types in a tissue were evaluated. Only positively stained cells are included in the table

In the urinary and reproductive organs, the most notable finding was a strong immunoreactivity in the epithelium of the proximal convoluted tubules of the kidney (Fig. 2a-c). Here, a moderate, homogenous cytoplasmic staining was found in addition to a stronger signal basolaterally. The immunoreactivity in the cells of the proximal tubules was clearly stronger than in the distal convoluted tubules (Fig. 2b). In the ovaries, an intense basolateral signal was observed in the granulosa cells of the follicles, while the cells of the corpus luteum showed a weaker, slightly granular signal from the cytoplasm (not shown). There was diffuse cytoplasmic staining in the epithelium of the endometrium and the endometrial glands (Fig. 3d). In the prostate, the secretory epithelium showed basolateral immunoreactivity, similar to the epithelium in the intestine, but there was also some diffuse cytoplasmic signal (Fig. 3e).

The bronchiolar epithelium in the lungs showed a cytoplasmic signal and a slightly increased intensity basolaterally. Around 10–20% of the cells had a marked increase in signal intensity compared to the others (Fig. 3f). These cells lacked cilia and had a slightly granulated cytoplasm, and were therefore assumed to be club cells. In lung tissue (Fig. 3f), as well as in the other tissues (not shown), the endothelial cells stained strongly.

**Fig. 4:**
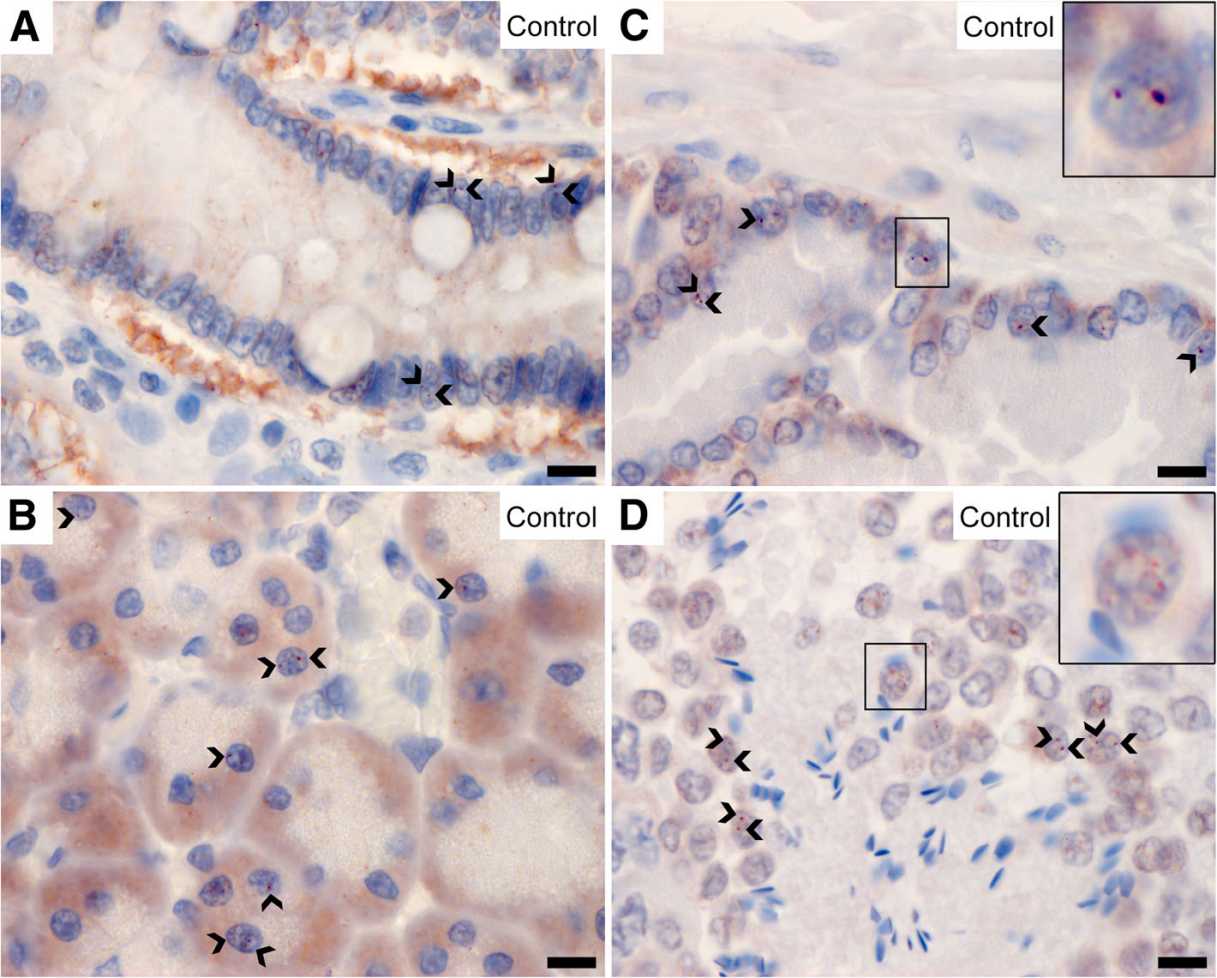
Centrosomal and nuclear localization of NDRG1. NDRG1 localized to one or two distinct structures (arrowheads) in the jejunal (**a**), pancreatic (**b**) and prostatic (**c**) epithelium, as well as the developing spermatogonia (**d**). The inset in C shows a magnified image of these punctate structures. Note that this signal is not present in all the cells. The inset in D shows a magnified image of the granular nuclear signal in the seminiferous tubules. Bar 10 μm

In some cells of the intestinal mucosa, the pancreas, the prostate, and the seminiferous tubules, one or two distinct structures close to, or overlying the nucleus, stained strongly (Fig. 4a-d). As described for the Schwann cell culture (see below), these punctate structures most probably represent centrosomes. Additionally, a fine granular nuclear signal was observed in some of these cells, most prominently in the seminiferous tubules. However, the centrosomal and nuclear signals were not present in all cells, suggesting that NDRG1 localizes to these structures at distinct phases of the cell cycle.

### Some NDRG1 expression was also observed in developing spermatids and mesenchymal cells

**Fig. 5:**
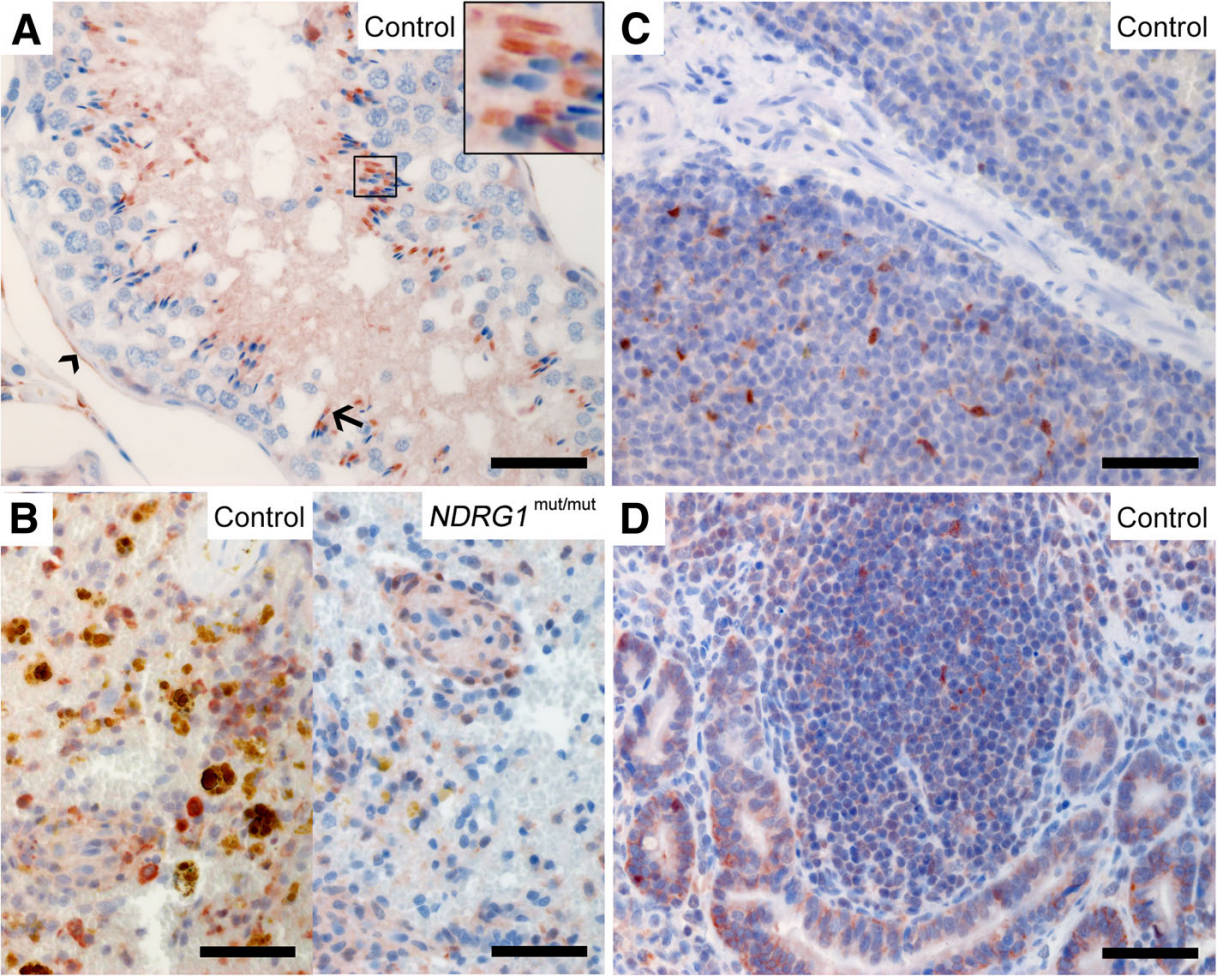
Expression of NDRG1 in developing spermatids and mesenchymal cells. NDRG1 is present in the midpiece (arrow and inset) of the spermatids and the myoepithelial cells (arrowhead) surrounding the seminiferous tubules (**a**), the ellipsoids and a subpopulation of the leukocytes in the spleen (**b**), follicles in the spleen (**c**) and the intestinal Peyer’s patches (**d**). The NDRG1 localization in the spleen of the *NDRG1*^mut/mut^ Alaskan malamute is similar to the control (**c**). Note the extensive hemosiderin deposits (yellow-brown granules) in the spleen of the control dog. Bar 50 μm

In the testis, as described, centrosomal and nuclear signals were present in the developing spermatogonia (Fig. 4d). Additionally, intense NDRG1 signals were observed in the developing spermatids in the testicle, localized to a short, circular structure in the midpiece of the spermatids (Fig. 5a). Moreover, NDRG1 was observed in myoepithelial cells surrounding the seminiferous tubules.

NDRG1 was also present in other mesenchymal cells. In lymphatic organs, NDRG1 was present in several cell types. In the spleen, there was signal in macrophages present in the wall of the ellipsoids (Fig. 5b), and a cytoplasmic staining in a subpopulation of leukocytes in the red pulp. In the subcapsular sinus and follicles of the lymph node cortex (not shown), the follicles (Fig. 5c) and periarteriolar lymphocyte sheaths of the spleen (not shown), as well as in the lymphoid tissue of the Peyer’s patches (Fig. 5d), dendritic cells, projecting interdigitating processes between surrounding lymphocytes, showed a prominent cytoplasmic and granular nuclear NDRG1 signal.

A weak and diffuse cytoplasmic NDRG1 staining was present in smooth muscle cells in both the intestinal and uterine wall, as well as in the wall of arterioles. A similar signal was observed in fibrocytes in several organs (not shown).

### NDRG1 was strongly expressed in Schwann cells

**Fig. 6:**
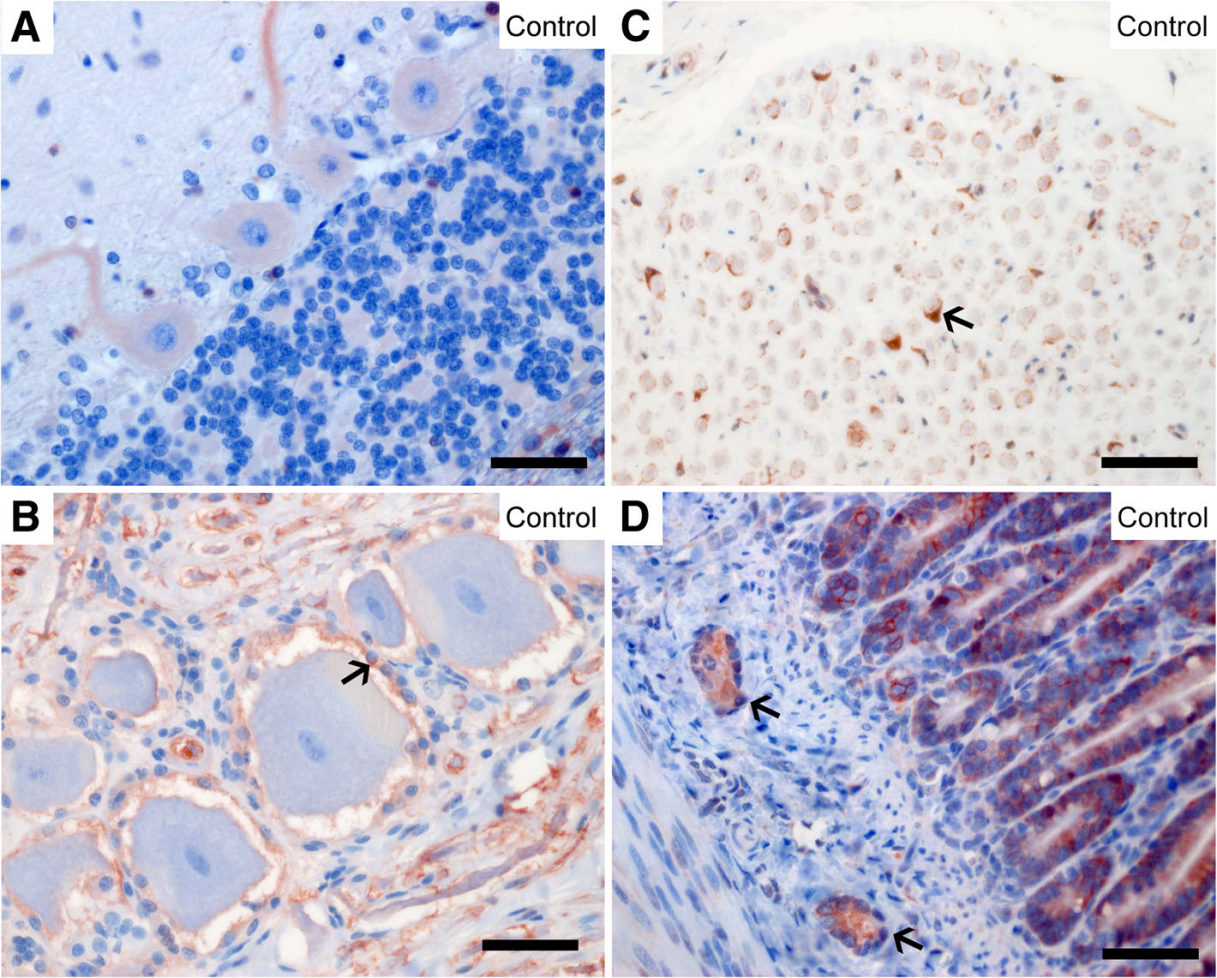
Expression of NDRG1 in the nervous system. NDRG1 is present in the Purkinje cells in the cerebellum (**a**), the satellite cells (arrow) surrounding the neurons in the dorsal root ganglia (**b**), the Schwann cells (arrow) in the peripheral nerves (**c**) and the submucosal nerve plexus of the enteric nervous system (**d**). Bar 50 μm

**Fig. 7:**
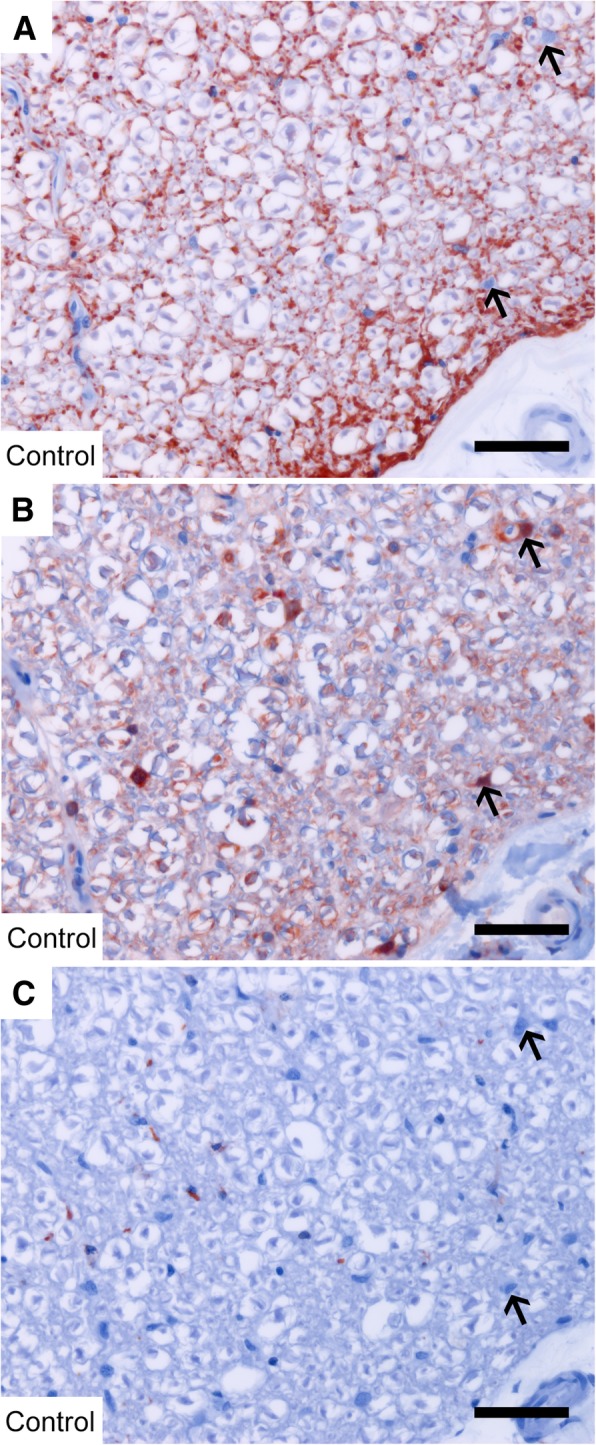
Immunohistochemistry against GFAP (**a**), NDRG1 (**b**) and Iba1 (**c**) on spinal cord white matter from control dogs Note that the NDRG1-positive cells (arrows) are not positive for GFAP or Iba1. Bar 50 μm

In the nervous system, NDRG1 was expressed in the cerebellar Purkinje cells (Fig. 6a), satellite cells surrounding the neurons in the dorsal root ganglia of the spinal cord (Fig. 6b), Schwann cells in the peripheral nerves (Fig. 6c), and the submucosal (Fig. 6d) and myenteric nerve plexus in the intestinal wall. There was no signal in neuronal cell bodies, nuclei (Fig. 6b) or axons (Fig. 6c). NDRG1 was also strongly expressed in the oligodendrocytes (Fig. 7b). Serial sections from the spinal cord labeled with the astrocyte-marker GFAP (glial fibrillary acidic protein, Fig. 7a), NDRG1 (Fig. 7b), and the microglia-marker Iba1 (ionized calcium binding adaptor molecule 1, Fig. 7c) showed that the NDRG1-positive cells are not positive for GFAP or Iba1, and therefore rather represent oligodendrocytes. In both oligodendrocytes and Schwann cells there were diffuse nuclear signals (Fig. 7b, 6c and 9a). In the ependymal cells, a cytoplasmic signal with increased staining intensity basolaterally was observed (not shown).

**Fig. 8:**
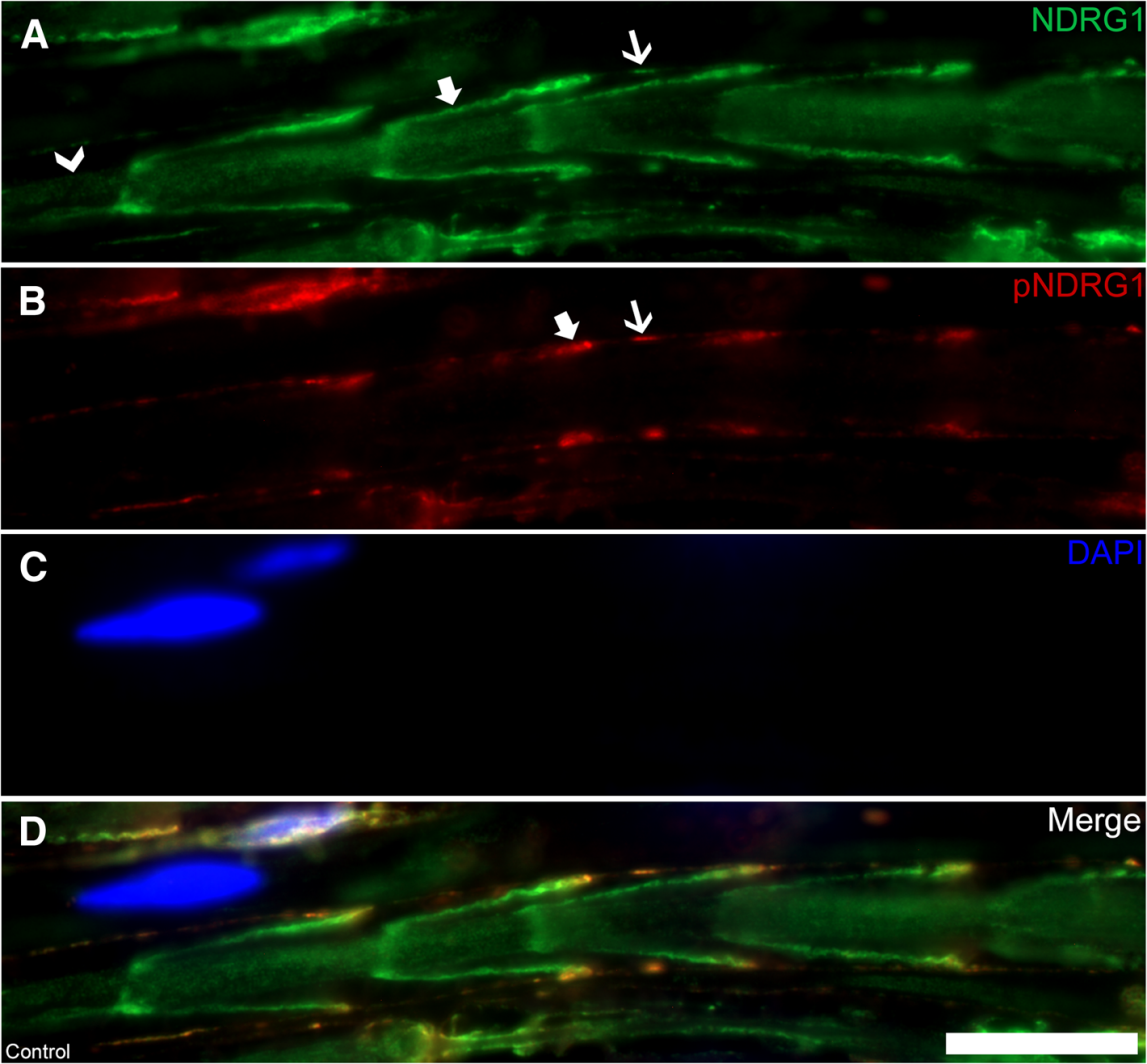
Immunofluorescence of total NDRG1 (green) and phosphorylated NDRG1 (Thr346) (red) in Schwann cell cytoplasm. Longitudinal (**a-d**) sections from a control dog. Total NDRG1 (**a**) is present in the adaxonal cytoplasm (arrowhead), the Schmidt-Lanterman clefts (bold arrow), and abaxonal cytoplasm (arrow), while pNDRG1 (**b**) is restricted to the outer parts of the Schmidt-Lanterman cleft (bold arrow) and abaxonal cytoplasm (arrow). DAPI (blue) labels nuclei (**c**). Bar 20 μm

**Fig. 9:**
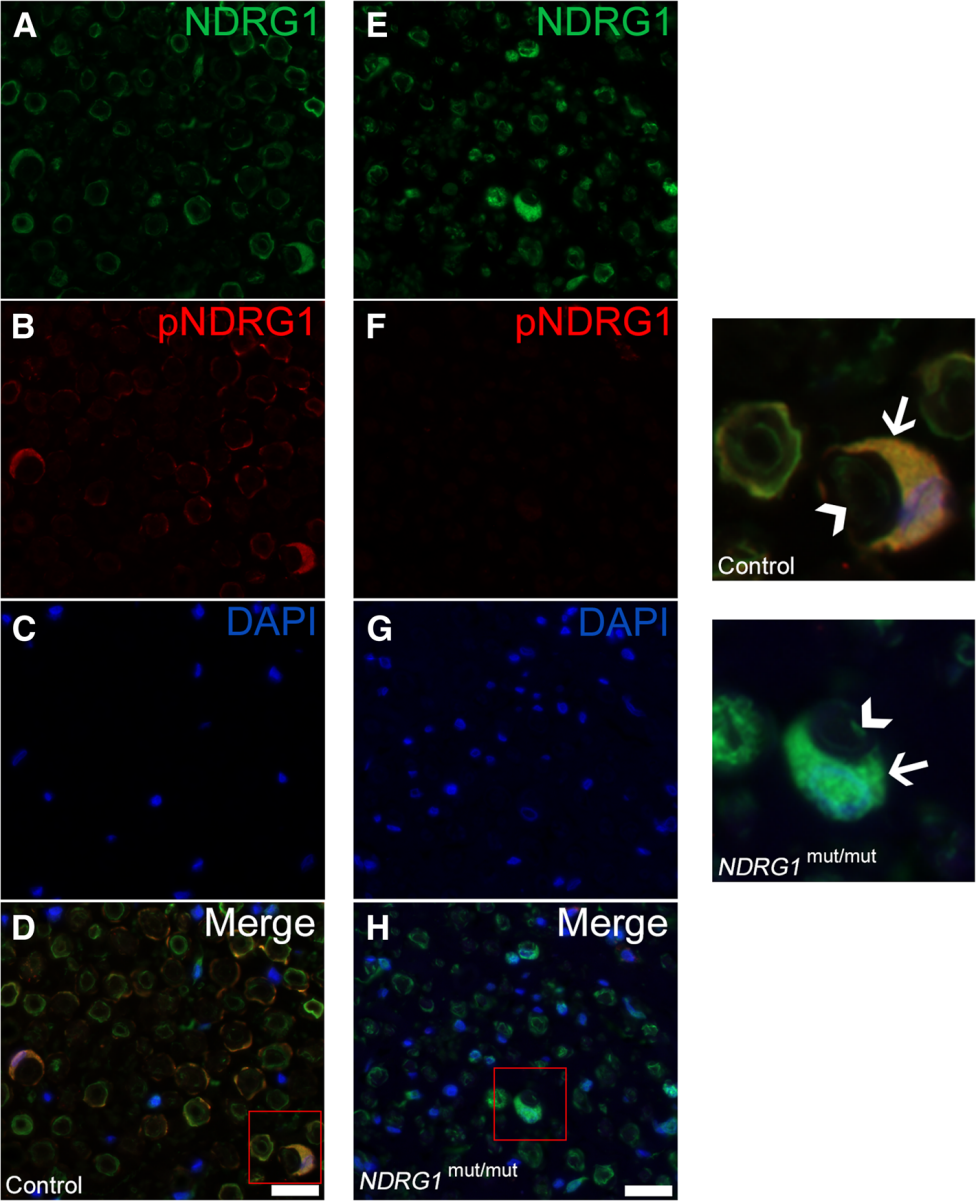
Immunofluorescence of total NDRG1 (green) and phosphorylated NDRG1 (Thr346) (red) in myelinating Schwann cells. In the peripheral nerve from an Alaskan malamute homozygous for the wild type *NDRG1* allele (**a-d**), strong pNDRG1 signal is present in the abaxonal cytoplasm. In comparison, in the nerve from the *NDRG1*^mut/mut^ Alaskan malamute (**e-h**), there is no pNDRG1 signal (**f**). In the magnified images, the adaxonal and abaxonal cytoplasm is indicated by arrowheads and arrows, respectively

To follow up on the strong expression of both total and phosphorylated NDRG1 in Western blots from peripheral nerve lysates, we further investigated the distribution of total NDRG1 and phosphorylated NDRG1 (pNDRG1 Thr346) in the peripheral nerves by immunofluorescence. Interestingly, while total NDRG1 (Fig. 8a) was present in both the adaxonal and abaxonal cytoplasm of the Schwann cells, as well as throughout the Schmidt-Lanterman clefts, phosphorylated NDRG1 (Fig. 8b) localized exclusively to the outer aspects of the Schmidt-Lanterman clefts and the abaxonal cytoplasm. In contrast, there was no detectable immunoreactivity in the compact myelin, neither against total nor phosphorylated NDRG1. In the nerve from the *NDRG1*^mut/mut^ Alaskan malamute, total NDRG1 was found both in the adaxonal and abaxonal Schwann cell cytoplasm (Fig. 9e), similar to the control (Fig. 9a). However, phosphorylated NDRG1 was absent (Fig. 9f).

### Phosphorylated NDRG1 was present in the nucleus of cultured, immature Schwann cells

**Fig. 10:**
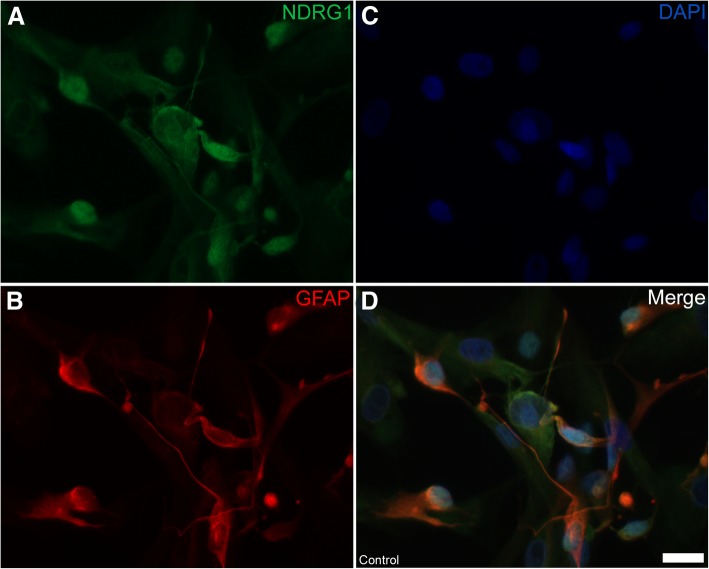
Expression of NDRG1 in cultured Schwann cells (**a-d**). Total NDRG1 (green) is present both in the cytoplasm and the nucleus of the immature, GFAP-positive Schwann cells (red). The Schwann cells have a characteristic spindle shape and a stronger NDRG1-expression than the GFAP-negative fibroblasts. Bar 20 μm

**Fig. 11:**
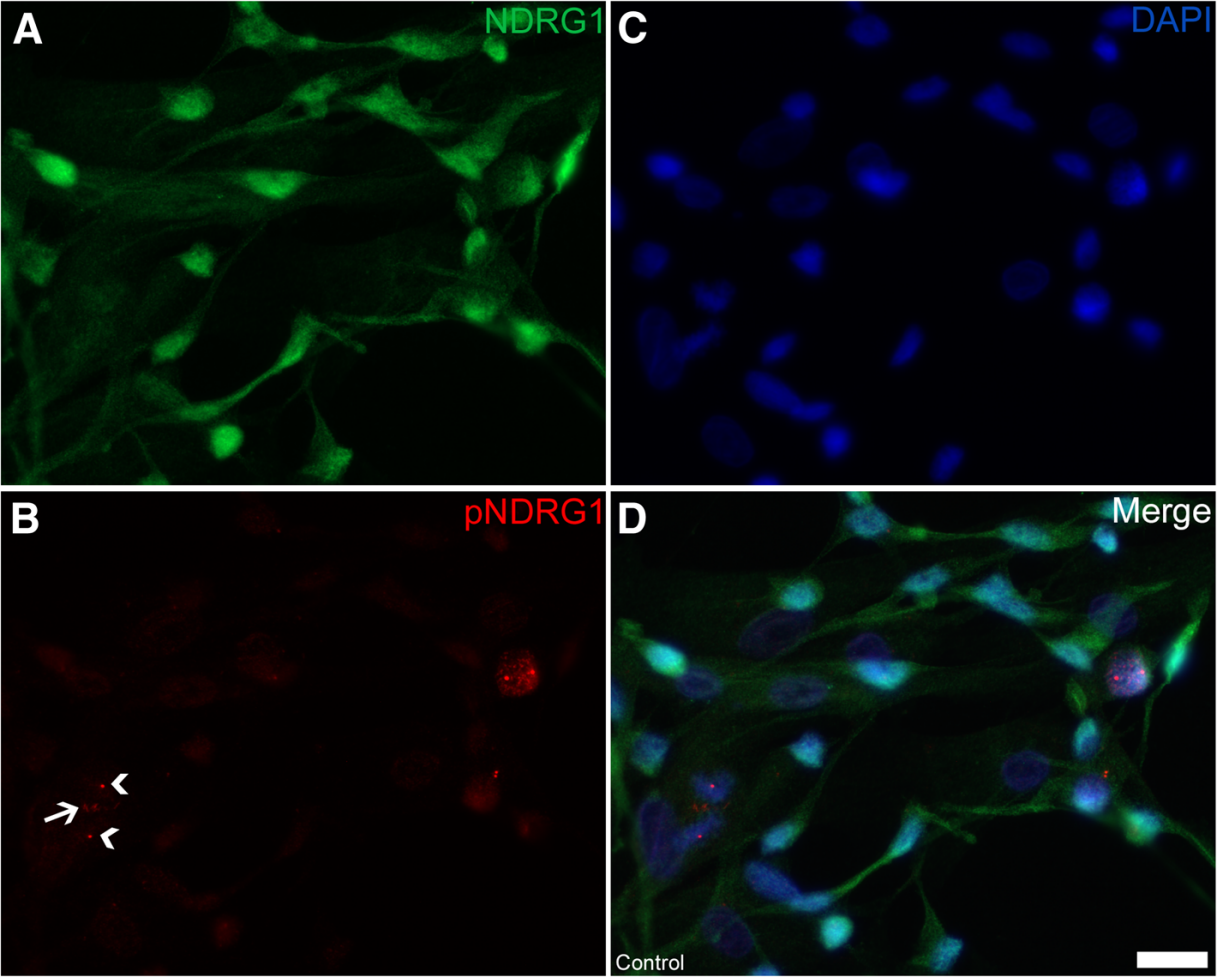
Expression of total NDRG1 and phosphorylated NDRG1 (Thr346) in cultured Schwann cells (**a-d**). While total NDRG1 (green) is present both in the cytoplasm and nucleus, pNDRG1 (red) localizes to the nucleus as well as two centrosome-like structures (arrowheads) and a midbody in a mitosis (arrow). Bar 20 μm

**Fig. 12:**
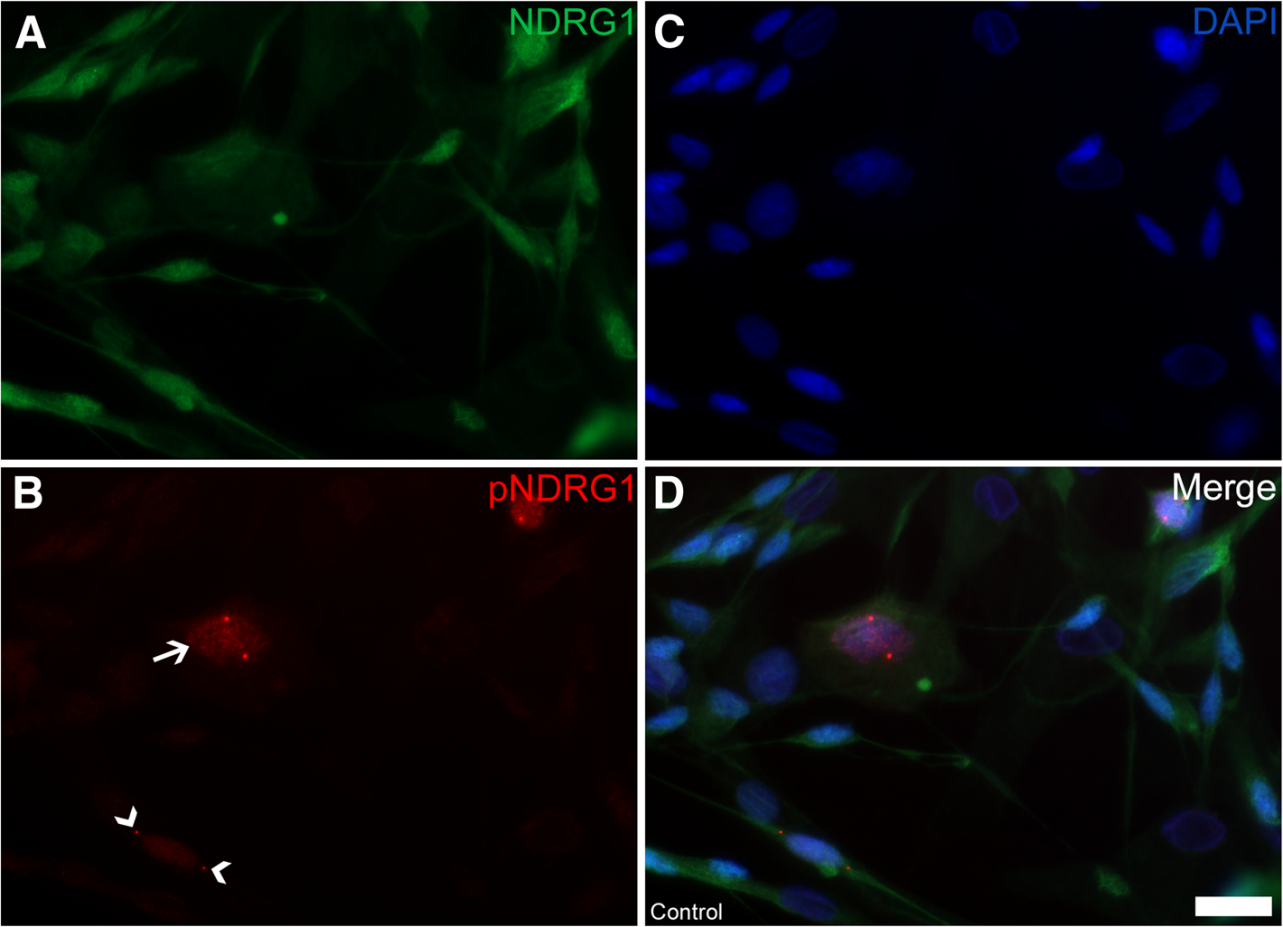
Expression of total NDRG1 (green) and pNDRG1 (Thr346) (red) in cultured Schwann cells (**a-d**). In some of the cells, there was a granular nuclear signal (arrow) (**b**). Even though the distinct, punctate structures were overlaying the nucleus in many instances, they are actually located in the cytoplasm (arrowheads) (**b**). Bar 20 μm

As there was a marked difference in the expression of the NDRG1 isoforms in Western blots from canine tissues, we next assessed the distribution of NDRG1 in primary Schwann cell culture. The cell culture consisted of canine Schwann cells and fibroblasts. Morphologically, the Schwann cells had a characteristic spindle shape, and stained strongly for GFAP (Fig. 10b). NDRG1 was present in both the Schwann cells and fibroblasts, albeit with a much weaker signal from the latter. In the cultured Schwann cells, total NDRG1 was present in both the cytoplasm and the nucleus (Fig. 11a). Whereas NDRG1 phosphorylated at Ser330 was present in both the cytoplasm and the nuclei of Schwann cells (not shown), similarly to total NDRG1, NDRG1 phosphorylated at Thr346 was primarily found in the nuclei (Fig. 11b). In some cells, punctate, juxta-nuclear structures, probably centrosomes, stained intensely for pNDRG1 Although these structures were often observed in the nuclear area, they were, in fact, localized in the cytoplasm close to the nuclear membrane (Fig. 12b). Additionally, in some of the cells a granular nuclear signal was observed (Fig. 12b).

## Discussion

Loss of NDRG1 functions causes degenerative polyneuropathy and increases malignancy of several human cancers. For instance, in colorectal cancer, NDRG1 counteracts EMT [14], thereby reducing metastatic potential. In both humans and dogs, specific mutations affecting *NDRG1* cause progressive polyneuropathies, classified as CMT4D in the former. Elucidating the normal subcellular localization and post-translational modifications of NDRG1 in diverse tissues holds one key to understanding its roles in both neuropathies and malignancies. Our data show that the subcellular localization of NDRG1 differs between canine tissues and that it varies dynamically through the cell cycle. Some of these fundamental features appear to be linked to post-translational modifications, such as phosphorylation. These observations also provide important clues as to how the cellular components, with which NDRG1 associates, exert their functions.

In this study, NDRG1 is detected in a variety of canine tissues, but most prominently in myelinating Schwann cells. The axons, however, appeared negative. In other organs, epithelial localization was mainly observed, as previously reported from human tissues [6]. However, there appears to be some marked differences between dogs and humans in the distribution of NDRG1. For example, no signal was detected in canine hepatocytes, but has been reported from human hepatocytes [6]. While we observed signal from canine mesenchymal cells, endothelia, and certain cells in the testicle and lymph nodes, no signal was observed in these tissues from humans by immunohistochemistry, although in testicle NDRG1 was detected by Western blotting [6]. Furthermore, all cell types in the human brain were negative [6], in contrast to the canine central nervous system where oligodendrocytes and Purkinje cells express NDRG1, a finding supported by Western blotting. Whereas epithelial cells mainly showed a prominent basolateral signal, NDRG1 had a more diffuse cytoplasmic distribution in the mesenchymal cells.

Western blot analysis revealed tissue-specific posttranslational modifications of NDRG1, including proteolytic processing. Studies of prostate cancer cells [18] and healthy kidney tissue [7] have identified truncated isoforms of NDRG1, with molecular masses varying from 35 to 40 kDa. Our data strongly resembles this, suggesting that these processing events are specific and functionally important. A proteolytic cleavage site between Cys49 and Gly50 has been suggested for prostate cancer cells [18] and would lead to an approximately 5 kDa decrease in the molecular mass of the protein. A detailed fragment analysis has not been performed here, however, we have identified strong expression of phosphorylated NDRG1 in the testicle, peripheral nerves, and Schwann cell culture. Clearly, the subcellular sorting and posttranslational processing of NDRG1 is complex and variable between tissues. Mutations in *NDRG1*, however, appear to solely precipitate pathology in the nervous system. This suggest that NDRG1 serves critically important, non-redundant roles in myelinating Schwann cells. Therefore, detailed studies of NDRG1 isoforms in Schwann cells are particularly important.

Based on our observation of high levels of phosphorylated NDRG1 in the peripheral nerves, this post-translational regulatory mechanism must be important for the physiological function of NDRG1 in nervous tissue. The NDRG1 protein is reported to be involved in several signaling pathways [25]. In the peripheral nerves, NDRG1 has been shown to be a target in the phosphoinositide 3-kinase (PI3-K)/Akt-pathway and is mainly phosphorylated by the serum and glucocorticoid-regulated kinase 1 (Sgk1) and Akt1 [20]. Although total NDRG1 was present throughout the cytoplasm of the myelinating Schwann cells, phosphorylated NDRG1 (Thr346) was restricted to the abaxonal cytoplasm of the control dogs. This finding suggests a phosphorylation-driven rerouting of the protein in Schwann cells. In mice, NDRG1 phosphorylation was shown to be dispensable for myelination in early life [20], but, to our knowledge, the importance of phosphorylated NDRG1 in peripheral nerves of animals later in life has not been addressed. The lack of phosphorylated NDRG1 in the nerve from the *NDRG1*^mut/mut^ Alaskan malamute could indicate that the mutation disrupts signaling in the Schwann cell. However, it could also be an unspecific result of the nerve pathology, so further studies are needed to elucidate the role of phosphorylated NDRG1 in the pathogenesis of neuropathies.

Although the predominant pathologic findings in dogs with *NDRG1*-associated neuropathies were reported to be axonal [4, 5], no NDRG1 signal was detected in the axons. This is in accordance with previous findings in peripheral nerves from humans and rodents [7]. As such, when our result of immunolocalization of NDRG1 to the Schwann cell cytoplasm is combined with the previously reported pathology of the polyneuropathies of Greyhounds and Alaskan malamutes, it seems that the expression of NDRG1 in Schwann cells is indispensable for both the Schwann cell and the axon. However, the fact that primary pathology in the axon leads to secondary changes in the Schwann cell, and vice versa, highlights the intimate relationship between the Schwann cell and the axon. The apparent divergence between the pathologic changes in dogs versus humans and rodents could, therefore, be more artefactual than real, and either caused by inter-species differences in the temporal progression of axonal atrophy [24] or the peripheral nerves being examined at different stages in the disease process.

Our observations of NDRG1 localizing to centrosomes, the midpiece of spermatids and basolateral cellular domains, clearly suggest that one or more isoforms of NDRG1 temporarily associate with microtubules or microtubule-organizing centers (MTOC). This is in accordance with a previous report, where NDRG1 was found to co-localize with gamma-tubulin in the centrosomes in colon cancer cells [21]. NDRG1 has been proposed to regulate centrosome number [26] and seems important for the formation of spindle fibers [27]. The midpiece of the spermatid contains the proximal and distal centrioles, and the latter extends distally as the axoneme [28]. The distal centriole degenerates during maturation of the spermatids and is not found in mature spermatozoa [28], which might explain why NDRG1 signal was not observed in all the seminiferous tubules. Although the basolateral NDRG1 signal in epithelia has been shown to originate from adherens junctions [6], this finding might actually support a tubulin-associated role for NDRG1, as the minus ends of the apico-basal microtubules are anchored to adherens junctions [29].

Microtubule-associated proteins (MAPs) bind directly to microtubules or tubulin via specific sequence elements, of which RSH is one [30]. Interestingly, the C-terminal tandem repeat of NDRG1 (GTRS**RSH**TSE) harbors this element. The tandem-repeat sequence is unique to NDRG1 in the NDRG family [31], and is repeated two and three times in the canine and human NDRG1 proteins, respectively. Therefore, we hypothesize that NDRG1 is a MAP that interacts with microtubules or tubulin through its C-terminal repeats. This enables NDRG1 to interact with other molecules through its N-terminal region, such as the phosphopantetheine attachment site and α/β hydrolase domain. The centrosomal signals in the Schwann cell cultures were only observed with the antibody against phosphorylated NDRG1 (Thr346). This phosphorylation site is located close to the putative microtubule-binding sequence of NDRG1 and could therefore affect the tubulin-binding properties of the protein. Phosphorylated NDRG1 is regulated through the cell cycle and has been suggested to play a role in microtubule organization and successful mitosis [21]. However, the exact mechanisms and functions of this phosphorylation, as well as interactions between NDRG1 and microtubules, remain to be clarified.

A granular nuclear signal was observed in the epithelium of the prostate, the pancreas, the intestinal crypts, dendritic cells in lymphatic tissues, spermatocytes, and cultured Schwann cells. These findings support the fact that NDRG1 shuttles between the cytoplasm and the nucleus, as previously reported from the epithelium of the prostate [6], in human trophoblasts exposed to hypoxia [32], and in Schwann cells during myelination [33]. The nuclear translocation of NDRG1 is puzzling, as no nuclear-targeting sequence has been identified in the protein [6]. Neither the nuclear nor centrosomal signal was present in all cells, suggesting that NDRG1 is redistributed during the cell cycle. In myelinating Schwann cells and oligodendrocytes, as well as cultured Schwann cells, a weak and diffuse nuclear staining was observed, indicating that NDRG1 is present in the nucleus in the G_0_ phase as well. During interphase and mitosis, NDRG1 concentrates at specific structures in the nucleus, centrosomes, and midbody.

## Conclusions

In conclusion, our results show a cell and context-dependent sorting of NDRG1. Our data from peripheral nerves and primary cultures of Schwann cells suggest that NDRG1 is highly dynamic in these cells, and most probably influenced by signaling events leading to reversible phosphorylation of the protein. Based on the lack of pNDRG1 signal in a nerve from the *NDRG1*^mut/mut^ Alaskan malamute, we propose that disease-causing mutations in *NDRG1* can disrupt signaling events in myelinating Schwann cells, leading to disturbance in the myelin homeostasis and axonal-glial cross talk, thereby precipitating polyneuropathy.

## Methods

### Animals

Samples were retrieved from the archive of the pathology laboratory, Norwegian University of Life Sciences. From control dogs, tissues from diseased organs were omitted from the analysis. Dogs of any breed, age, and gender were included. Alaskan Malamute dogs included as controls in the analyses were homozygous for the wild type *NDRG1* allele, while the neuropathic Alaskan malamute was homozygous for the *Gly98Val* mutation in *NDRG1*. Details on the individuals are summarized in Table 2.

**Table 2:** Signalment of the individuals included in the analyses of NDRG1 in canine tissues and cells

Breed	Age	Gender	Analysis
Spitz dog	3 weeks	Female	IHC
Norwegian Lundehund	2 months	Male	WB
Labrador retriever	8 months	Female	IHC
French Bulldog	6 years	Male	IHC
Alaskan malamute	7 years	Male	IHC
Norwegian Elkhound	7 years	Male	IHC, IF
Alaskan malamute	8 years	Female	IHC, IF, Schwann cell culture, WB
Alaskan malamute	11 years	Female	IHC
Gordon setter	12 years	Female	IHC
Pointer	12 years	Male	WB
English Springer Spaniel	14 years	Male	IHC
Alaskan malamute, homozygous for *Gly98Val* mutation	10 years	Female	IHC, IF

*IHC* Immunohistochemistry, *IF* Immunofluorescence, *WB* Western blot

### Tissue sampling

Tissues from the following organs were sampled shortly after pentobarbital-euthanasia; skeletal muscle, myocardium, lung, liver, colon, jejunum, pancreas, spleen, lymph node, kidney, uterus, ovary, testicle, prostate, peripheral nerves, cerebellum, cerebrum and spinal cord. Samples for immunohistochemistry were fixed in 10% buffered formalin and subsequently paraffin embedded. Samples for Western blotting were snap frozen in isopentane, transferred to liquid nitrogen, and stored at − 80 °C until analysis.

### Antibodies

Five different antibodies against NDRG1 were used in the analyses; Mouse monoclonal anti-NDRG1 antibody (catalog number WH0010397, Sigma-Aldrich, Merck, Darmstadt, Germany), goat polyclonal anti-NDRG1 antibody (catalog number PA5–18109, Invitrogen, Thermo Fisher Scientific, Massachusetts, United States), rabbit polyclonal anti-NDRG1 antibody (catalog number HPA006881, Sigma-Aldrich, Merck), rabbit monoclonal phospho-specific anti-NDRG1 (Thr346) (catalog number 5482, Cell Signaling Technology, Leiden, Netherlands), and a rabbit monoclonal phospho-specific anti-NDRG1 (Ser330) (catalog number ab124713, Abcam, Cambridge, United Kingdom). Additionally, antibodies against glial fibrillary acidic protein (GFAP) (catalog number Z0334, Dako, California, United States) and Iba1 (catalog number 019–19741, Fujifilm Wako Chemicals, Neuss, Germany) were used as markers for glial cells. Details on the antibodies are summarized in Table 3.

**Table 3:** Antibodies and dilutions used in the analyses of NDRG1 in canine tissues and cells

Name	Catalog number	Producer	Dilutions		
			IHC	IF	WB
Monoclonal anti-NDRG1 antibody produced in mouse	WH0010397	Sigma-Aldrich, Merck, Darmstadt, Germany	1/2000	1/2000	1/2000
Polyclonal anti-NDRG1 antibody produced in goat	PA5–18109	Invitrogen, Thermo Fisher Scientific	1/750	NA	NA
Polyclonal anti-NDRG1 antibody produced in rabbit	HPA006881	Sigma-Aldrich, Merck	1/100	1/100	1/1000
Monoclonal phospho-specific anti-NDRG1 (Thr346) produced in rabbit	5482	Cell Signaling Technology, Leiden, Netherlands	NA	1/500	1/1000
Monoclonal phospho-specific anti-NDRG1 (Ser330) produced in rabbit	ab124713	Abcam, Cambridge, United Kingdom	NA	1/75	1/10000
Anti-glial fibrillary acidic protein	Z0334	Dako, California, United States	1/500	1/500	NA
Anti-Iba1	019–19,741	Fujifilm Wako Chemicals, Neuss, Germany	1/250	NA	NA

*IHC* Immunohistochemistry, *IF* Immunofluorescence, *WB* Western blot, *NA* Not analyzed

### Western blotting

The samples were thawed, and the nerve tissue was freed from the epineurial fat. The tissue samples were lysed in homogenization buffer (50 mM Tris HCl, 150 mM NaCl, 1 mM EDTA, 0.25% DOC, 1% NP40, pH 7.4) supplemented with protease inhibitor cocktail (Roche complete, Roche Holding AG, Basel, Switzerland) and anti-phosphatase (Halt™ Phosphatase Inhibitor Cocktail, Thermo Fisher Scientific). Protein concentrations were measured using Protein assay (Bio-Rad, Hercules, California, United States).

25 μg protein from individual samples were separated by sodium dodecyl sulfate (SDS) polyacrylamide gel electrophoresis (12% Criterion™ XT-Bis-Tris, Bio-Rad), and transferred to polyvinylidene fluoride (PVDF) membranes (GE Healthcare, Little Chalfont, United Kingdom). The membranes were blocked with 5% non-fat milk in TBS-Tween for 90 min at room temperature and incubated with primary antibodies diluted in blocking buffer overnight at 4 °C. Thereafter, the membranes were washed and incubated for 90 min in 1% non-fat milk containing alkaline phosphatase-conjugated anti-mouse IgG (dilution 1/4000, Thermo Fisher Scientific) or anti-rabbit IgG (dilution 1/4000, GE Healthcare). The membrane was developed using EFC™ substrate (GE Healthcare) and visualized with Typhoon 9200 (Amersham Bioscience, GE Healthcare).

Immunoprecipitation from peripheral nerve lysate was performed with Dynabeads® Protein G (Novex, Life Technologies, Thermo Fisher Scientific) according to the manufacturer’s instructions. Three μg of anti-NDRG1 antibody (catalog number HPA006881, Sigma-Aldrich, Merck) was used for the precipitation. Western blotting was subsequently performed as previously described with another anti-NDRG1 antibody (catalog number WH0010397, Sigma-Aldrich, Merck).

### Immunohistochemistry

Sections of 3–4 μm were placed on glass slides (Superfrost Plus®, Menzel Gläser, Thermo Fisher Scientific) and stored at 4 °C until staining. The slides were deparaffinized in xylene and rehydrated through a descending alcohol series. Sections were washed in PBS for 5 min, twice between each step, except before incubation with the primary antibody. For all antibodies except anti-Iba1, antigen retrieval was performed by heating the slides for 15 min in citrate buffer (0.01 M, pH 6.0) in a Lab Vision™ PT Module (Thermo Fisher Scientific). For anti-Iba1, the slides were incubated with trypsin (1 mg/mL) in Tris HCl-buffer (0.1 M, pH 8.0) with 0.1% CaCl_2_ for 40 min at 37 °C. Endogenous peroxidase activity was blocked with 3% H_2_O_2_ in methanol for 10 min. Non-specific antibody binding was blocked by incubating the slides for 30 min in 5% bovine serum albumin (BSA) with 2% normal serum from the same species as the secondary antibody. The sections were incubated with three different primary antibodies against NDRG1, diluted in 1% BSA for 60 min at concentrations summarized in Table 3. To optimize the signal intensity and signal-to-noise ratio, a modified protocol was used for the rabbit polyclonal anti-NDRG1 antibody; the slides were blocked with 1% normal serum in PBS, the primary antibody was diluted in PBS, while the secondary antibody was diluted in PBS with 2% normal serum.

Next, the sections were incubated with biotinylated secondary antibodies (dilution 1/50, catalog number BA-9200, BA-9500 and BA-1000, Vector Laboratories, California, United States) diluted in 1% BSA for 30 min. The sections were subsequently incubated with Vectastain Elite ABC reagent (Vectastain Elite ABC Kit, Vector Laboratories) for 30 min and then with ImmPact AEC Peroxidase Substrate (catalog number SK-4205, Vector Laboratories) for 3 min. In analyses where Iba1 was included, secondary antibodies conjugated to horseradish peroxidase-labeled polymer and AEC + -substrate from the EnVision+ kit (catalog number K4009, Dako) were used. The sections were counterstained with hematoxylin and mounted with Aquatex (Merck, Darmstadt, Germany). Sections where the primary antibodies were omitted were used as negative controls. For each tissue, samples from at least two individuals were included in the analysis. Micrographs were taken using an Axio Imager 2 microscope equipped with an Axiocam 506 color camera (Zeiss, Oberkochen, Germany).

### Immunofluorescence on paraffin sections of peripheral nerves

Peripheral nerves were fixed and processed as described previously. Antigen retrieval was performed by heating the slides in citrate buffer (0.01 M, pH 6.0) in a microwave. The temperature in the solution was held at 92 °C for 5 min, thereafter the slides were kept in the hot solution for another 15 min before being rinsed in PBS. Non-specific antibody activity was blocked by incubating the slides for 30 min in 5% BSA with 2% normal serum from the same species as the secondary antibody. The sections were incubated with primary antibodies diluted in 1% BSA for 60 min at concentrations summarized in Table 3. The slides were rinsed three times for 5 min in PBS and incubated in secondary antibodies diluted in 1% BSA for 30 min. The secondary antibodies used were goat anti-mouse IgG Alexa Fluor 488 (dilution 1/400, catalog number A-11029, Invitrogen, Thermo Fisher Scientific) and goat anti-rabbit IgG Alexa Fluor 594 (dilution 1/400, catalog number A-11006, Invitrogen, Thermo Fisher Scientific). The slides were rinsed three times for 5 min in PBS and mounted with ProLong Gold Antifade Mountant with 4′,6 diamidino-2-phenyl indole (DAPI; Molecular Probes, Thermo Fisher Scientific). Slides where the primary antibodies were omitted were used as negative controls. Micrographs were taken using an Axio Imager 2 microscope equipped with an Axiocam 506 mono camera (Zeiss).

### Schwann cell cultures

A primary Schwann cell culture from a control dog was established as previously described [34]. Briefly, an approximately 5 cm long whole trunk biopsy of the common fibular nerve was sampled shortly after pentobarbital-euthanasia of a dog. Immediately, the epineurium was removed and the fibers teased apart. Thereafter, the fibers were subjected to enzymatic digestion by 0.25% dispase and 0.05% type I collagenase in Dulbecco’s Modified Eagle Medium (DMEM). For expansion of the cell cultures, complete medium (high glucose-DMEM supplemented with 10% FBS, 1% GlutaMAX, 1% penicillin/streptomycin, 25 μg/mL gentamicin, 10 nM neuregulin and 2 μM forskolin) was used. For cryopreservation, the cells were trypsinated, resuspended in complete medium, centrifugated and frozen in Recovery Cell Culture Freezing Medium.

### Immunolabeling of cultured Schwann cells

The cell cultures were grown in eight well-glass chambered slides in complete medium. The cells were rinsed twice in PBS, fixed in 2% paraformaldehyde for 10 min at room temperature, rinsed in PBS three times for 1 min, permeabilized with 0.2% Triton X-100 in PBS and rinsed in PBS three times for 1 min. The cells were incubated 30 min in a blocking solution consisting of 2% BSA, 0.2% TWEEN 20, 7% glycerol and 2% goat serum. Thereafter, the cells were incubated with primary antibodies diluted in blocking solution for 60 min at room temperature at concentrations summarized in Table 3. The cells were rinsed three times for 1 min in PBS and incubated with secondary antibodies for 30 min. The secondary antibodies and mounting medium were the same as used for immunofluorescence on paraffin sections. Wells where the primary antibodies were omitted were used as negative controls.

## Abbreviations

BSA: Bovine serum albumin
CMT4D: Charcot-Marie-Tooth type 4D
Cys49: Cysteine at amino acid position 49
DMEM: Dulbecco’s Modified Eagle Medium
EMT: Epithelial-mesenchymal transition
GFAP: Glial fibrillary acidic protein 1
Gly50: Glycine at amino acid position 50
*Gly98Val*: Glycin to valine substitution at amino acid position 98
Iba1: Ionized calcium binding adaptor molecule 1
IF: Immunofluorescence
IHC: Immunohistochemistry
LDL: Low-density lipoprotein
MAP: Microtubule-associated proteins
MTOC: Microtubule-organizing center
*NDRG1*: *N-myc downstream regulated gene 1*
PBS: Phosphate-buffered saline
PI3K: Phosphoinositide 3-kinase
pNDRG1: Phosphorylated NDRG1
PVDF: Polyvinylidene fluoride
SDS: Sodium dodecyl sulfate
Ser330: Serine at amino acid position 330
Sgk1: Serum and glucocorticoid-regulated kinase 1
TBS: Tris-buffered saline
Thr346: Threonine at amino acid position 346
WB: Western blotting
